# Amino Acid at Position 166 of NS2A in Japanese Encephalitis Virus (JEV) Is Associated with In Vitro Growth Characteristics of JEV

**DOI:** 10.3390/v12070709

**Published:** 2020-06-30

**Authors:** Shigeru Tajima, Satoshi Taniguchi, Eri Nakayama, Takahiro Maeki, Takuya Inagaki, Chang-Kweng Lim, Masayuki Saijo

**Affiliations:** Department of Virology I, National Institute of Infectious Diseases, 1-23-1 Toyama, Shinjuku, Tokyo 162-8640, Japan; rei-tani@nih.go.jp (S.T.); nakayama@nih.go.jp (E.N.); tomaeki@nih.go.jp (T.M.); tinagaki@nih.go.jp (T.I.); ck@nih.go.jp (C.-K.L.); msaijo@nih.go.jp (M.S.)

**Keywords:** Japanese encephalitis virus, genotype V, NS2A, reverse genetics

## Abstract

We previously showed that the growth ability of the Japanese encephalitis virus (JEV) genotype V (GV) strain Muar is clearly lower than that of the genotype I (GI) JEV strain Mie/41/2002 in murine neuroblastoma cells. Here, we sought to identify the region in GV JEV that is involved in its low growth potential in cultured cells. An intertypic virus containing the NS1-3 region of Muar in the Mie/41/2002 backbone (NS1-3^Muar^) exhibited a markedly diminished growth ability in murine neuroblastoma cells. Moreover, the growth rate of a Muar NS2A-bearing intertypic virus (NS2A^Muar^) was also similar to that of Muar in these cells, indicating that NS2A of Muar is one of the regions responsible for the Muar-specific growth ability in murine neuroblastoma cells. Sequencing analysis of murine neuroblastoma Neuro-2a cell-adapted NS1-3^Muar^ virus clones revealed that His-to-Tyr mutation at position 166 of NS2A (NS2A^166^) could rescue the low replication ability of NS1-3^Muar^ in Neuro-2a cells. Notably, a virus harboring a Tyr-to-His substitution at NS2A^166^ (NS2A^Y166H^) showed a decreased growth ability relative to that of the parental virus Mie/41/2002, whereas an NS2A^Muar^-based mutant virus, NS2A^Muar-H166Y^, showed a higher growth ability than NS2A^Muar^ in Neuro-2a cells. Thus, these results indicate that the NS2A^166^ amino acid in JEV is critical for the growth and tissue tropism of JEV in vitro.

## 1. Introduction

Japanese encephalitis virus (JEV) is a mosquito-borne arbovirus that causes the serious neurological disorder Japanese encephalitis (JE). JE represents a major public health problem in Asia, with an estimated 68,000 cases of JE per year resulting in 15,000 fatalities, mostly in children [1,2,3]. JEV belongs to the genus *Flavivirus* in the family *Flaviviridae* and is amplified in a bird– and pig–mosquito transmission cycle [4]. The mosquitoes transmit JEV to dead-end host humans.

JEV harbors a single-stranded, positive-sense RNA genome that encodes three structural proteins (C, prM, and E) and seven nonstructural proteins (NS1, NS2A, NS2B, NS3, NS4A, NS4B, and NS5) from a single open reading frame, and the genome also contains noncoding regions (NCRs) at its 5′- and 3′-terminal ends. Based on genomic RNA homology, JEV strains are classified into five genotypes: GI, GII, GIII, GIV, and GV [5,6]. GI and GIII strains have been the most widely distributed strains in JE-endemic areas since the 1990s [7,8]; GII strains constitute the third most common group and have been detected in Indonesia, Singapore, South Korea, Malaysia, and Australia [9]; GIV strains have been isolated only in Indonesia [9]; and GV strains also represent an extremely rare group—the first GV JEV strain (Muar) was isolated from a patient with encephalitis in Malaysia in 1952, but then no other GV JEV isolates were identified for >50 years [6]. However, in 2009, an infectious GV JEV strain (XZ0934) was isolated from a *Culex* mosquito pool in China [10], and a GV JEV strain was also detected in Korea in 2010 [11]. In Korea, GI, GIII, and GV JEV strains have been endemic in recent years, but GV is currently the main genotypic strain circulating in Korea [12,13]. Furthermore, GV JEV was isolated from a JE patient in 2015 [14].

JE vaccines currently available are derived from GIII strains. These JE vaccines were previously reported to potentially exhibit less ability to induce neutralizing antibodies against GV JEV than those against other JEV genotypes [15,16]. Conversely, IgGs raised against GV JEV XZ0934 were reported to show a poor neutralizing ability against GIII JEV [17]. These findings raise the possibility that GV JEV is distinct from other JEV genotypes in terms of antigenicity, and the current GIII-derived JE vaccines might thus provide inadequate levels of protection against GV JEV. The low identity in amino acid sequences between GV and GIII JEV could be one reason for the poor efficacy of the GIII-derived vaccines against GV JEV [16]. Therefore, attention must be paid to the dynamics of the circulating JEV strains in JE-endemic areas.

The characteristics of GV JEV remain poorly understood because only a few GV JEV strains have been isolated to date and only limited studies have been performed on these strains. We previously showed that the neuroinvasiveness of GV JEV Muar in mice was substantially higher than that of GI JEV Mie/41/2002 [16]. Moreover, it was shown that a recombinant GV JEV of the XZ0934 strain exhibited higher neuroinvasiveness in mice than recombinant GIII JEV, and that the structural protein region (C-prM-E) of GV JEV plays a critical role in the increased pathogenicity of GV JEV [17]. We also reported that the E and prM proteins of GV JEV are responsible for the highly virulent characteristics of GV JEV in a mouse model [18].

The Muar strain also presents unique features in in vitro growth. The growth ability of Muar in mouse neuroblastoma cells was found to be clearly lower than that of Mie/41/2002 [16,18]. Our findings also showed that the 5’-NCR-C-prM-E region of Muar, which is responsible for the high neuroinvasiveness of the strain, does not influence the growth characteristics of Muar in murine neuroblastoma cells [18]. Thus, our previous data indicate that the growth properties of JEV in mouse neuronal cells do not necessarily reflect pathogenicity in mice. However, the growth characteristics of Muar in vitro is of great interest in understanding the tissue tropism of JEV. To gain insight into the nature of Muar in in vitro growth, we sought to identify the region in GV JEV responsible for its low growth ability in cultured cells, particularly in murine neuroblastoma cells. Thus, we generated recombinant intertypic chimeric and missense mutant JEV strains in the backbone of the GI JEV strain Mie/41/2002 using a reverse-genetics system that we previously established, and then analyzed the mechanism for the growth properties of these JEV strains in vitro.

## 2. Materials and Methods

### 2.1. Cell Culture

African green monkey kidney Vero cells (strain 9013) and mouse neuroblastoma Neuro-2a cells were cultured at 37 °C in 5% CO_2_ in Eagle’s minimal essential medium (MEM) (Sigma-Aldrich, St. Louis, MO, USA) supplemented with 10% heat-inactivated fetal bovine serum (FBS) (Sigma-Aldrich) and 100 U/mL penicillin-streptomycin (Thermo Fisher Scientific, Waltham, MA, USA). Mouse neuroblastoma N18 cells were cultured at 37 °C in 5% CO_2_ in Dulbecco’s modified Eagle’s medium (Sigma-Aldrich) supplemented with 10% FBS and 100 U/mL penicillin-streptomycin.

### 2.2. Viruses

The GI JEV strain Mie/41/2002 (GenBank accession no. AB241119), which was isolated from pig serum in Japan in 2002 [19,20], and the GV JEV strain Muar (GenBank accession no. HM59272), which was isolated from a patient with encephalitis in Malaysia in 1952, were used [16]. The working virus stocks were prepared through amplification in Vero cells.

### 2.3. Recombinant Viruses

**Figure 1:**
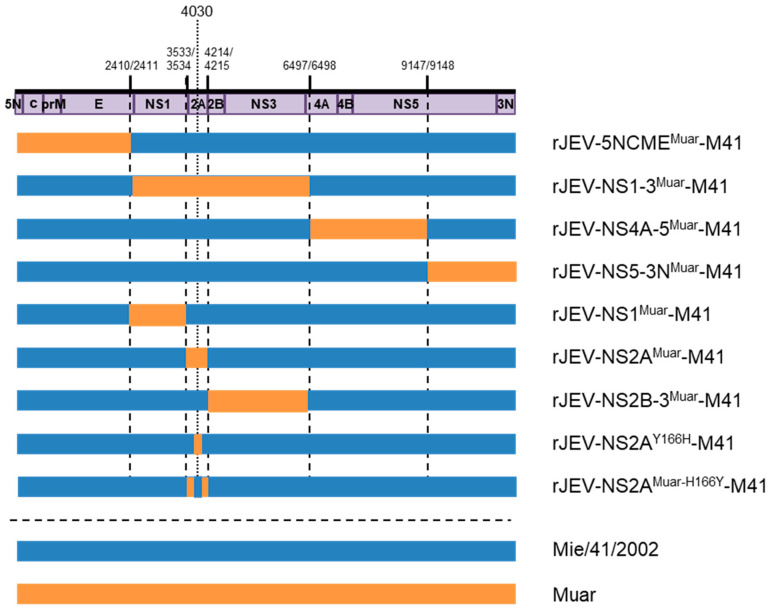
Schematic representation of the genomic structure of mutant Japanese encephalitis viruses (JEVs) used in this study. Regions derived from Mie/41/2002 and Muar strains are shown in blue and orange, respectively; numbers: nucleotide positions in the Mie/41/2002 genome.

Four JEV molecular clones of GI-GV intertypic and missense mutant viruses (rJEV-5NCME^Muar^-M41 (5NCME^Muar^), rJEV-NS1-3^Muar^-M41 (NS1-3^Muar^), rJEV-NS4A-5^Muar^-M41 (NS4A-5^Muar^), and rJEV-NS5-3N^Muar^-M41 (NS5-3N^Muar^)) described previously were used [18]. We constructed the following mutant viruses: rJEV-NS1^Muar^-M41 (NS1^Muar^), rJEV-NS2A^Muar^-M41 (NS2A^Muar^), rJEV-NS2B-3^Muar^-M41 (NS2B-3^Muar^), rJEV-NS2A^Y166H^-M41 (NS2A^Y166H^), and rJEV-NS2A^Muar-H166Y^-M41 (NS2A^Muar-H166Y^) in the Mie/41/2002 backbone, as described previously [16,20]. Briefly, the distinct genomic regions of the infectious cDNA clone rJEV(Mie/41/2002)/pMW119 were replaced with the corresponding regions of the Muar genome (Figure 1). The cDNA fragments of Muar were amplified through one-step RT-PCR performed using a PrimeScript II High-Fidelity RT-PCR kit (Takara Bio, Shiga, Japan). The primers are listed in Supplementary Table S1. Each of the amplified fragments was inserted into the corresponding region by using conventional molecular cloning methods and the In-Fusion Cloning system (Takara Bio). The single missense mutations, NS2A^Y166H^ and NS2A^Muar-H166Y^, were introduced into the strains rJEV(Mie/41/2002)/pMW119 and rJEV-NS2A^Muar-H166Y^-M41/pMW119, respectively, by means of inverse-PCR-based site-directed mutagenesis [21]. The nucleotide sequences of the viral genome region of the recombinant clones were determined after amplification of the plasmids in *Escherichia coli* STBL2 (Thermo Fisher Scientific). The recombinant viruses were recovered by transfecting Vero cells with in vitro-transcribed recombinant viral RNA, as previously described [21]. An aliquot of the culture supernatant of the Vero cells transfected was passaged once in Vero cells, and the culture fluid was used as the recombinant virus solution (v1). The nucleotide sequences of the recombinant viruses were also determined; no additional nucleotide mutations were detected.

### 2.4. Plaque-Formation Assay and Analysis of Growth Kinetics

Infectious viral titers for each sample were determined using a plaque-formation assay; for the assay, Vero cells (~3 × 10^5^/well) were plated in 12-well plates and inoculated with each of the viruses for 1 h at 35–37 °C. Next, an MEM-based overlay medium containing 1% methyl cellulose and 2% FBS was added to the wells, and the cells were incubated for 5 days at 35–37 °C, after which the cells were fixed using a 10% formalin-PBS solution and stained with methylene blue, as described previously [21].

The growth of the JEV strains was analyzed as described previously [20]. Briefly, cells were cultured in 6-well culture plates and infected with each JEV strain in MEM supplemented with 2% FBS (2F/MEM) at a multiplicity of infection (MOI) of 0.1 plaque-forming units (PFU)/cell. Small aliquots of the media were collected at one-day intervals, and the infectious viral titers were determined using the plaque-formation assay in Vero cells, as described above.

### 2.5. Adaptation of Virus to Mouse Neuroblastoma Cells

NS1-3^Muar^ (v1) was inoculated into Neuro-2a cells (in 6-well plates) at an MOI of 0.1 PFU/cell, and the cells were incubated for 3 days at 35–37 °C. The culture supernatant (p1) was recovered and a small aliquot (200 μL) was used for the next inoculation into Neuro-2a cells in 6-well plates, which were then incubated for 3 days at 35–37 °C. The passaging was repeated ten times (p1 to p10).

### 2.6. Plaque Cloning (Limiting-Dilution Method)

NS1-3^Muar^ p10 solution was diluted to 5 PFU/mL with 2F/MEM; the diluted virus solution (50 μL = 0.25 PFU) was added to Vero cells cultured in 12-well plates, and the cells were incubated at 35–37 °C for 6–7 days. Culture supernatants were recovered and used in the plaque-formation assay to assess the plaque sizes of the viruses and to measure the infectious titer in the solutions, as described above.

### 2.7. Statistical Analysis

Viral titers for each sample determined by plaque-forming assay as described above were statistically compared by using BellCurve for Excel (Social Survey Research Information, Tokyo, Japan). Data were presented as mean ± standard deviation of the three independent experiments. Significance was analyzed using the two-way ANOVA test.

## 3. Results

### 3.1. Growth of GI-GV Intertypic Mutants 5NCME^Muar^, NS1-3^Muar^, NS4A-5^Muar^, and NS5-3N^Muar^ in Mouse Neuroblastoma Cells

**Figure 2:**
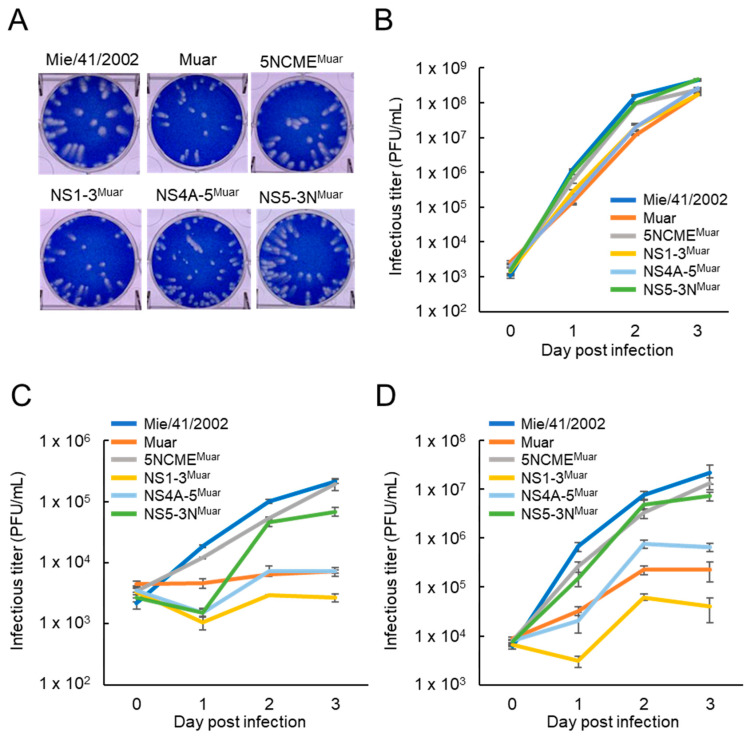
(**A**) Plaque phenotypes of Mie/41/2002, Muar, and four intertypic strains, rJEV-5NCME^Muar^-M41 (5NCME^Muar^), rJEV-NS1-3^Muar^-M41 (NS1-3^Muar^), rJEV-NS4A-5^Muar^-M41 (NS4A-5^Muar^), and rJEV-NS5-3N^Muar^-M41 (NS5-3N^Muar^), in Vero cells. (**B**–**D**) Growth curves measured for Mie/41/2002, Muar, 5NCME^Muar^, NS1-3^Muar^, NS4A-5^Muar^, and NS5-3N^Muar^ in Vero (**B**), N18 (**C**), and Neuro-2a (**D**) cells. Cells were plated into 6-well culture plates and infected with the JEV strains at a multiplicity of infection (MOI) of 0.1 PFU/cell. Values: mean ± standard deviation from three independent experiments.

To identify the genomic region in Muar responsible for its low growth ability in cultured cells, the growth kinetics of four Mie/41/2002 background intertypic mutant viruses incorporated with the partial Muar gene, 5NCME^Muar^, NS1-3^Muar^, NS4A-5^Muar^, and NS5-3N^Muar^ (Figure 1), were investigated in Vero cells and in two murine neuroblastoma cell lines (N18 and Neuro-2a) (Figure 2). To construct NS4A-5^Muar^ and NS5-3N^Muar^ mutants, the NS5 region was split into methyltransferase (NS4A-5^Muar^) and RNA-dependent RNA polymerase (NS5-3N^Muar^) domains. The plaques formed by NS1-3^Muar^, NS4A-5^Muar^, and NS5-3N^Muar^ were slightly smaller than those formed by the parental virus Mie/41/2002, but were of a similar size as the plaques formed by Muar (Figure 2A). The growth kinetics of 5NCME^Muar^ and NS5-3N^Muar^ in Vero, N18, and Neuro-2a cells were similar to those of Mie/41/2002, and these were slightly higher than those measured for Muar, NS1-3^Muar^, and NS4A-5^Muar^ (Figure 2B–D). Although the growth properties of NS1-3^Muar^ and NS4A-5^Muar^ in these cells were similar to those of Muar, NS1-3^Muar^ exhibited the lowest growth ability in the murine neuroblastoma cells (Figure 2C,D). The results suggested that the regions NS1-3 and NS4A-5 are related to the low growth capacity of Muar in cultured cells.

### 3.2. Growth of GI-GV Intertypic Mutants NS1^Muar^, NS2A^Muar^, and NS2B-3^Muar^ in Mouse Neuroblastoma Cells

**Figure 3:**
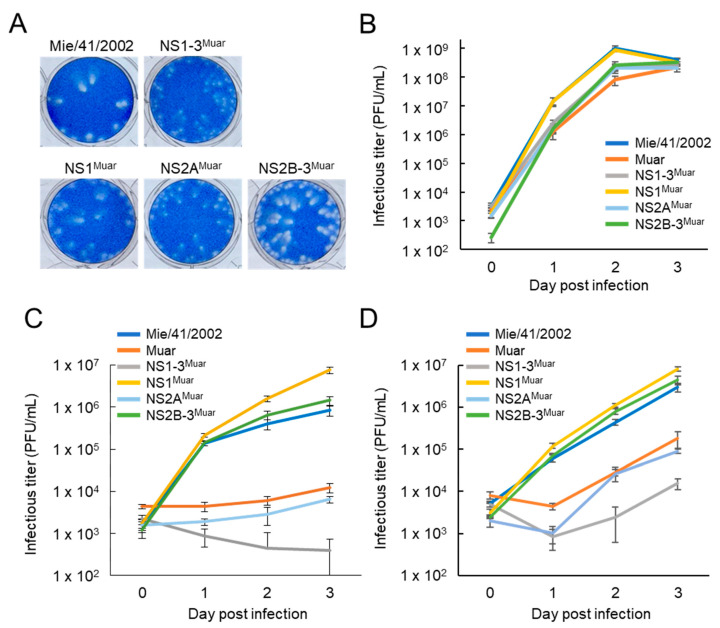
(**A**) Plaque phenotypes of Mie/41/2002 and four intertypic strains, NS1-3^Muar^, rJEV-NS1^Muar^-M41 (NS1^Muar^), rJEV-NS2A^Muar^-M41 (NS2A^Muar^), and rJEV-NS2B-3^Muar^-M41 (NS2B-3^Muar^), in Vero cells. (**B**–**D**) Growth curves measured for Mie/41/2002, Muar, NS1-3^Muar^, NS1^Muar^, NS2A^Muar^, and NS2B-3^Muar^ in Vero (**B**), N18 (**C**), and Neuro-2a (**D**) cells. Cells were plated into 6-well culture plates and infected with the JEV strains at an MOI of 0.1 PFU/cell. Values: mean ± standard deviation from three independent experiments.

We next focused on the NS1-3 region of Muar, which more potently affected the growth of Mie/41/2002 in N18 and Neuro-2a cells than the NS4A-5 region (Figure 2C,D). For the analysis, we generated three novel intertypic mutants, NS1^Muar^, NS2A^Muar^, and NS2B-3^Muar^ (Figure 1). The plaques generated by NS2A^Muar^ were smaller than the plaques of Mie/41/2002, NS1^Muar^, and NS2B-3^Muar^ but were similar in size to those of NS1-3^Muar^ (Figure 3A). Furthermore, the growth kinetics of NS2A^Muar^ were also similar to those of Muar and NS1-3^Muar^ in N18 and Neuro-2a cells (Figure 3C,D). By contrast, Mie/41/2002 and NS1^Muar^ showed similar growth rates in Vero, N18, and Neuro-2a cells, and the growth rates of NS2B-3^Muar^ were also similar to those of Mie/41/2002 in the murine neuroblastoma cells (Figure 3B–D). The results suggested that the substitutions of the NS1-3 and NS2A regions of Mie/41/2002 with the respective regions from Muar decreased the growth ability of Mie/41/2002 in cultured cells.

### 3.3. Adaptation of NS1-3^Muar^ to Mouse Neuroblastoma Neuro-2a Cells

**Figure 4:**
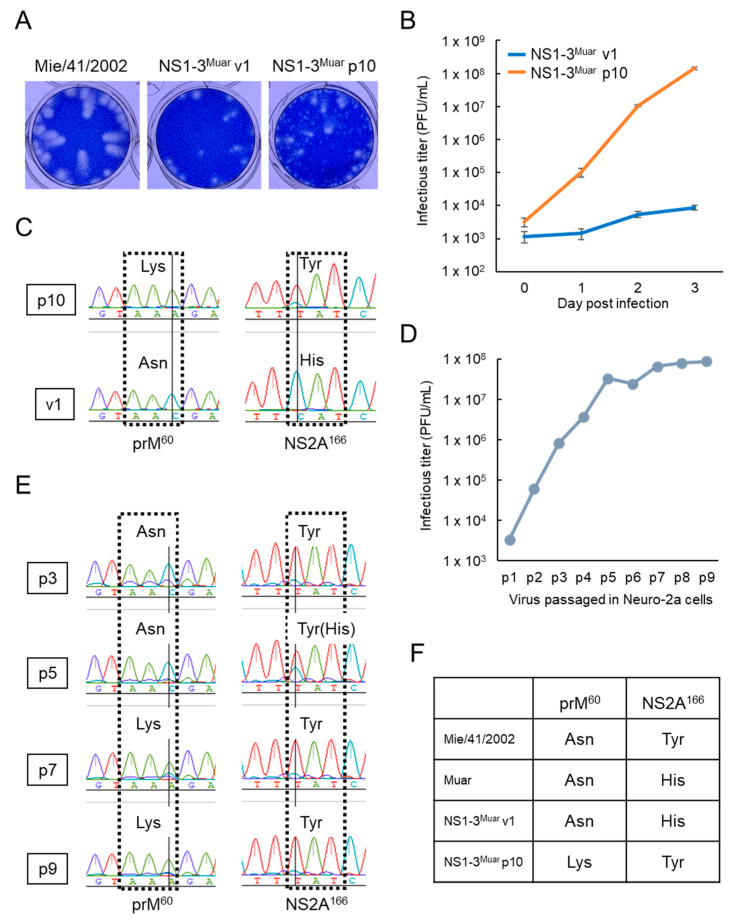
(**A**) Plaque phenotypes of Mie/41/2002, original NS1-3^Muar^ virus v1 (NS1-3^Muar^ v1), and Neuro-2a-adapted NS1-3^Muar^ virus p10 (NS1-3^Muar^ p10) in Vero cells. (**B**) Growth curves measured for NS1-3^Muar^ v1 and NS1-3^Muar^ p10 in Neuro-2a cells. Cells were plated into 6-well culture plates and infected with the JEV strains at an MOI of 0.1 PFU/cell. Values: mean ± standard deviation from three independent experiments. (**C**) Sequencing electrograms of nucleotides at amino acid positions 60 of prM (left peaks) and 166 of NS2A (right peaks) in NS1-3^Muar^ v1 (lower peaks) and NS1-3^Muar^ p10 (upper peaks). (**D**) Infectious titers of NS1-3^Muar^ viruses passaged in Neuro-2a cells. The infectious titer of each virus (p1 to p9) was determined using Vero cells. (**E**) Sequencing electrograms of nucleotides at amino acid positions 60 of prM (left peaks) and 166 of NS2A (right peaks) in NS1-3^Muar^ p3, p5, p7, and p9. (**F**) Amino acid residues at positions 60 of prM and 166 of NS2A in Mie/41/2002, Muar, and NS1-3^Muar^ v1 and p10.

To search for the site critical for the reduction of growth potency in murine neuroblastoma cells, we generated a mutant NS1-3^Muar^ adapted to Neuro-2a cells by continuously passaging NS1-3^Muar^ in Neuro-2a cells ten times (p10) (Figure 4A). The growth rate of this virus, NS1-3^Muar^ p10, was clearly higher than that of the original virus, NS1-3^Muar^ v1, which indicated that NS1-3^Muar^ p10 acquired a higher replication ability in Neuro-2a cells (Figure 4B). The complete nucleotide sequence of the NS1-3^Muar^ p10 genome was determined, revealing that there were two differences in the nucleotide sequence, one was located in the prM region and the other was located in NS2A region, between NS1-3^Muar^ v1 and NS1-3^Muar^ p10 (Figure 4C). The two nucleotide mutations identified in NS1-3^Muar^ p10 were also accompanied by amino acid substitutions, Asn-to-Lys at position 60 of prM (prM^60^) and His-to-Tyr at position 166 of NS2A (NS2A^166^), which suggested that these amino acid alterations could be associated with acquiring the increased growth ability of NS1-3^Muar^ p10 in Neuro-2a cells. Intriguingly, the infectious titers of the viruses passaged in Neuro-2a cells increased clearly between the once-passaged virus (p1) and the 5-times-passaged virus (p5), which implied that the virus adapted to Neuro-2a cells in the early stage of repeated passaging (Figure 4D). Thus, we determined the nucleotide sequences of the positions corresponding to prM^60^ and NS2A^166^ in NS1-3^Muar^ p3, p5, p7, and p9 (Figure 4E); this revealed that the Asn-to-Lys substitution at the nucleotide level in prM^60^ of NS1-3^Muar^ occurred between p5 and p7, whereas the His-to-Tyr substitution in NS2A^166^ occurred before p3. The amino acid residue at prM^60^ was Asn in Mie/41/2002, Muar, and NS1-3^Muar^ v1 but Lys in NS1-3^Muar^ p10 (Figure 4F and Supplementary Figure S1), whereas the NS2A^166^ residue was His in Muar and NS1-3^Muar^ v1 but Tyr in NS1-3^Muar^ p10 and Mie/41/2002 (Supplementary Figure S2). These results suggest that the His-to-Tyr mutation in NS1-3^Muar^ p10 is mainly associated with the adaptation of the virus to Neuro-2a cells.

### 3.4. Characterization of Neuro-2a-Adapted NS1-3^Muar^ p10 Clones

**Figure 5:**
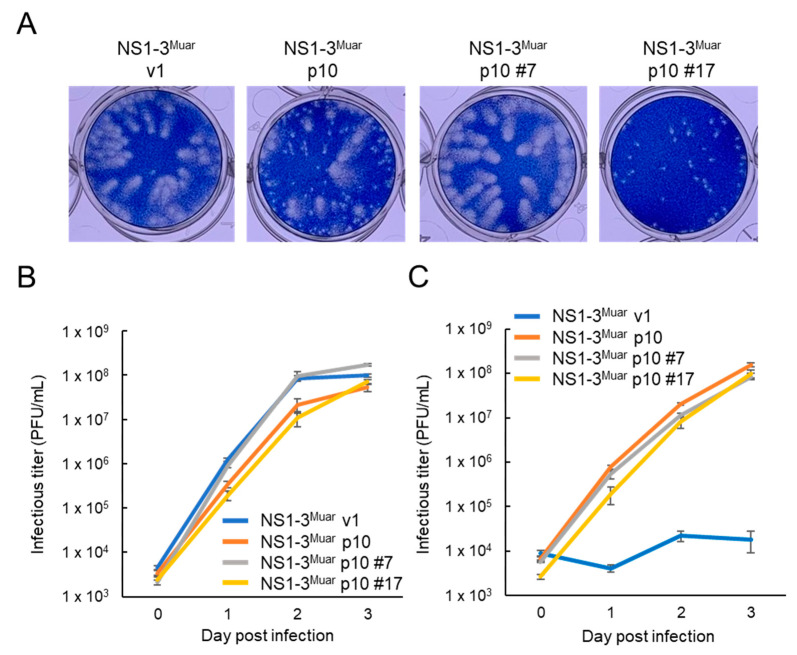
(**A**) Plaque phenotypes of NS1-3^Muar^ v1, NS1-3^Muar^ p10, and NS1-3^Muar^ p10 clones #7 (NS1-3^Muar^ p10#7) and #17 (NS1-3^Muar^ p10#17) in Vero cells. (**B**,**C**) Growth curves measured for NS1-3^Muar^ v1, NS1-3^Muar^ p10, NS1-3^Muar^ p10#7, and NS1-3^Muar^ p10#17 in Vero (**B**) and Neuro-2a (**C**) cells. Cells were plated into 6-well culture plates and infected with the JEV strains at an MOI of 0.1 PFU/cell. Values: mean ± standard deviation from three independent experiments.

The results of plaque assays performed using Vero cells suggested that NS1-3^Muar^ p10 includes at least two distinct types of viruses: native-sized-plaque and pin-sized-plaque viruses (Figure 4A and Figure 5A). Small plaques were observed with NS1-3^Muar^ p7 and p10 viruses but not p3 and p5 viruses (Supplementary Figure S3). To clarify the nucleotide sequence of the native-sized-plaque and pin-sized-plaque variants, single-clone viruses were obtained from the NS1-3^Muar^ p10 solution using a limiting-dilution method. Five pin-sized-plaque and native-sized-plaque clones were obtained, and the nucleotide sequences of the prM and NS2A regions of the clones were determined (Table 1). All five clones that formed native-sized plaques and 4/5 clones that formed pin-sized plaques featured an amino acid change at NS2A^166^ (His-to-Tyr), whereas 4/5 clones that formed pin-sized plaques and only 1–2/5 clones that formed native-sized plaques featured a change at prM^60^ (Asn-to-Lys). We also found an additional amino acid substitution (Gln to Arg) at position of 7 of prM (prM^7^) in all five native-sized-plaque clones, but this alteration was not detected in NS1-3^Muar^ p10 and the pin-sized-plaque clones. To further examine the characteristics of these clones, we selected two clones: pin-sized-plaque clone #17 and native-sized-plaque clone #7 (Table 1 and Figure 5A). NS1-3^Muar^ p10 and pin-sized-plaque clone #17 grew slightly slower than NS1-3^Muar^ v1 and native-sized-plaque clone #7 in Vero cells (Figure 5B). By contrast, NS1-3^Muar^ p10 and both selected clones, #7 and #17, showed a clearly increased replication ability in Neuro-2a cells as compared with that of NS1-3^Muar^ v1 (Figure 5C). The results indicate that the His-to-Tyr substitution at NS2A^166^ in NS1-3^Muar^ p10 is critical for the efficient growth of NS1-3^Muar^ in Neuro-2a cells and also suggest that the Tyr residue at NS2A^166^ in Mie/41/2002 plays a key role in acquiring the high replication ability of this strain in murine neuroblastoma cells.

### 3.5. Growth of Site-Directed Mutants NS2A^Y166H^ and NS2A^Muar-H166Y^ in Mouse Neuroblastoma Cells

**Figure 6:**
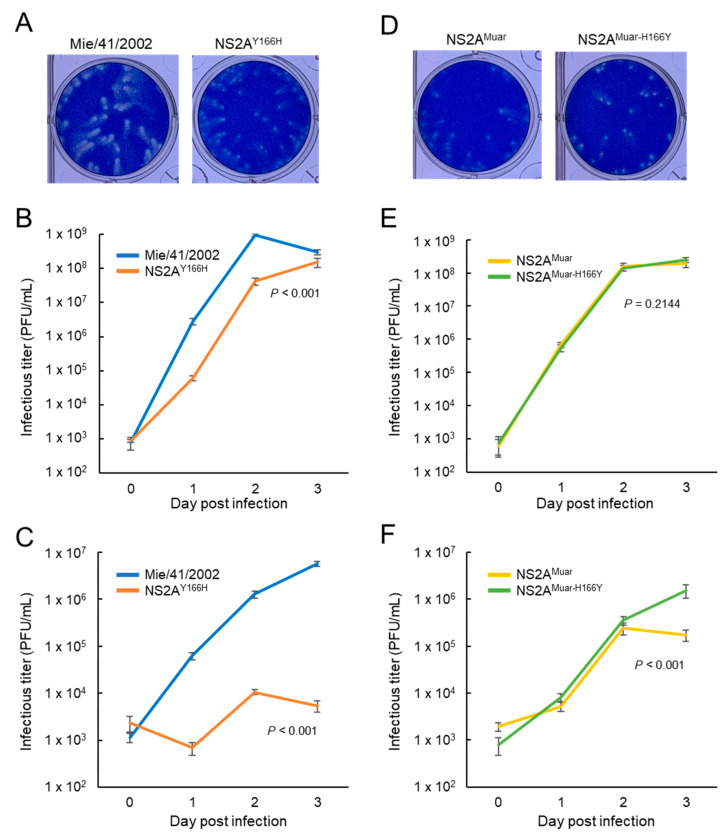
(**A**) Plaque phenotypes of Mie/41/2002 and site-directed mutant rJEV-NS2A^Y166H^-M41 (NS2A^Y166H^). (**B**,**C**) Growth curves measured for Mie/41/2002 and NS2A^Y166H^ in Vero (**B**) and Neuro-2a (**C**) cells. Cells were plated into 6-well culture plates and infected with the JEV strains at an MOI of 0.1 PFU/cell. Values: mean ± standard deviation from three independent experiments. Significance was analyzed using the two-way ANOVA test. (**D**) Plaque phenotypes of NS2A^Muar^ and its site-directed mutant rJEV-NS2A^Muar-H166Y^-M41 (NS2A^Muar-H166Y^). (E and F) Growth curves measured for NS2A^Muar^ and NS2A^Muar-H166Y^ in Vero (**E**) and Neuro-2a (**F**) cells. Cells were plated into 6-well culture plates and infected with the JEV strains at an MOI of 0.1 PFU/cell. Values: mean ± standard deviation from three independent experiments. Significance was analyzed using the two-way ANOVA test.

To investigate whether the Tyr residue at NS2A^166^ in GI Mie/41/2002 is crucial for the high growth potency of this strain in mouse neuroblastoma cells, we generated a recombinant Mie/41/2002 strain harboring a Tyr-to-His substitution at NS2A^166^, NS2A^Y166H^ (Figure 1 and Figure 6A). NS2A^Y166H^ grew slightly slower than Mie/41/2002 in Vero cells (Figure 6B), and the growth of the mutant was also strikingly slower than that of Mie/41/2002 in Neuro-2a cells (Figure 6C). Moreover, we produced an additional site-directed mutant, JEV NS2A^Muar-H166Y^, and investigated how this mutation influences the growth of NS2A^Muar^ in vitro (Figure 6D–F). NS2A^Muar-H166Y^ showed higher growth ability than NS2A^Muar^ in Neuro-2a cells, although the two viruses exhibited nearly equal growth kinetics in Vero cells. These results suggest that NS2A^166^ affects the growth of JEV in murine neuroblastoma cells.

## 4. Discussion

The GV strain Muar shows lower growth potential in murine neuronal cells than GI Mie/41/2002 [16]. This study was conducted to identify the genomic region that is related to this difference in in vitro growth property between the strains. It was shown that NS2A of Muar was one of the regions responsible for the Muar-specific lower growth ability in murine neuroblastoma cells. Furthermore, sequencing analysis of revertant viruses derived from NS1-3^Muar^ revealed that His-to-Tyr substitution at position 166 of NS2A^Muar^ rescued the low replication ability of the NS1-3^Muar^ mutant. Thus, the results indicate that the NS2A^166^ amino acid in JEV influences the growth and tissue tropism of JEV in vitro. Although previous study indicates that amino acid sequences in the E protein are involved in the adaptation of JEV in Neuro-2a cells [22], no differences in E region amino acid sequences were found between NS1-3 ^Muar^ v1 and the NS1-3^Muar^ p10. An Asn-to-Lys mutation at prM^60^ was found in NS1-3^Muay^ p10, which may be associated with the appearance of the pin-sized plaque variants. In Neuro-2a cells, the growth rate of NS1-3^Muar^ p10 #17 clone, with Lys at prM^60^, was similar to that of NS1-3^Muar^ p10 #7 clone, with Asn at prM^60^ (Figure 5C). Therefore, it appears that the mutation at prM^60^ is not primarily involved in the increased growth ability of NS1-3^Muar^ p10 in Neuro-2a cells. However, pin-sized viruses were a major component of the p10 (Figure 4), implying that the prM^60^ mutation in NS1-3^Muar^ may confer an advantage in propagating NS1-3^Muar^ in Neuro-2a cells over continuous passages. Flavivirus NS2A is a highly hydrophobic transmembrane protein that localizes in the endoplasmic reticulum (ER) membrane and interacts with virus genomic RNA and other structural and nonstructural viral proteins [23,24,25,26,27]. NS2A has several functions throughout the virus life cycle and pathogenesis: viral RNA synthesis [23,25,28], virion assembly and secretion [25,26,27,28,29,30,31], escape from host immunity [32,33,34,35,36], and pathogenicity in vitro and in vivo [37,38,39]. On the other hand, the cellular factor tripartite motif-containing protein 25 (TRIM52) interacts with and degrades JEV NS2A, which results in the inhibition of JEV replication [40]. Furthermore, the nucleotide sequence of the JEV NS2A-coding region is also involved in the expression of the NS1’ protein [41,42]. In dengue virus NS2A, the N-terminal 68 amino acids are located in the ER lumen, the core region (amino acids 69 to 209) integrally spans the ER membrane, and the C-terminal tail (amino acids 210 to 218) is located in the cytosol [43]. The results of previous studies and our result of SOSUI (classification and secondary structure prediction system for membrane proteins) analysis indicate that JEV NS2A contains eight predicted transmembrane segments (pTMS1–8) (Supplementary Figure S2) [43,44].

The growth of the NS1-3^Muar^ mutant, which exhibited poor replication ability in murine neuroblastoma cells, was rescued when an additional mutation was introduced in the NS2A region (NS2A^H166Y^). The results suggest that NS2A^166^ is one of the sites responsible for the replication efficiency of the virus in mouse neuroblastoma cells. The NS2A^Y166H^ substitution in Mie/41/2002 also drastically decreased its growth ability in Neuro-2a cells (~1 × 10^3^-fold) (Figure 6C), but the degree of change in growth rate between NS2A^Muar^ and NS2A^Muar-H166Y^ was low (~1 × 10^1^-fold) (Figure 6F). These results imply that factors other than NS2A^166^ including other sites in Muar NS1-3 and cellular factors are associated with the decreased growth potential of Muar.

A single amino acid substitution, Phe-to-Leu substitution at position 113 in NS2A (NS2A^113^) of GIII JEV affects viral growth in Neuro-2a cells [44]. The amino acid residue at NS2A^113^ in Mie/41/2002 and Muar is Phe, and the residue at NS2A^113^ also appears to be conserved in GI, GIII, and GV JEVs (Supplementary Figure S2); thus, NS2A^113^ does not likely contribute to the disparity in growth ability demonstrated in Mie/41/2002 and Muar. However, NS2A of JEV may play a key role in the efficient replication of JEV in murine neuronal cells [44]. The NS1-to-NS3 region of Muar is not associated with the high-virulence phenotype of Muar, which indicates that the growth property of NS2A-associated mutants in mouse neuroblastoma cells is unrelated to the virulence of Muar in mice [18]. The Phe-to-Leu or Leu-to-Phe substitutions at NS2A^113^ in GIII JEV strains does not affect their pathogenicity in mice, which supports the notion that virus propagation in vitro does not directly reflect virus replication and pathogenicity in vivo. The pathology caused by JEV infection is complex, and numerous factors—replication of the virus and invasion of the immune cells, cytokines, and chemokines in the brain—are involved in the pathogenicity of JEV.

Flavivirus NS2A has been comprehensively analyzed genetically and biochemically [24,26,27,28,39,43,45]. Zheng et al. conducted systematic alanine-scanning mutagenesis of Zika virus NS2A and classified the phenotypes of the mutants into five groups [27]. An Arg-to-Ala mutation in Zika virus NS2A (NS2A^R171A^), in which the mutation site is near JEV NS2A^166^, belongs to a group of mutants defective for virion assembly, and this suggests that NS2A^166^ in JEV may also be associated with the virion assembly of JEV. Alanine-scanning mutagenesis was also performed in dengue virus NS2A, and a Leu-to-Phe mutation in NS2A (NS2A^L181F^) was identified to act as a compensatory mutation in several NS2A mutants bearing alanine substitutions [28,39]. JEV NS2A^166^ is located in pTMS7 (amino acids 164 to 183) (Supplementary Figure S2), and dengue virus NS2A^181^ is also located in pTMS7, which corresponds to pTMS7 of JEV NS2A. The findings for dengue virus NS2A and the results in this study suggest that pTMS7 interacts intramolecularly with other pTMSs of NS2A and regulates the function of NS2A in virus replication [28,39].

Dengue virus NS2A pTMS7 (amino acids 165 to 186) traverses the ER membrane, whereas Zika virus NS2A pTMS6 (amino acids 152 to 185) interacts peripherally with the ER membrane [27,43]. A recent study showed that dengue virus NS2A cannot trans-complement a virion assembly-defective NS2A mutant of Zika virus [45]. These findings suggest that different flavivirus NS2A molecules feature distinct membrane topologies and that each pTMS functions in a virus-type-specific manner. Thus, the findings in dengue virus and Zika virus NS2A might not be readily applicable to other flavivirus NS2A molecules. Clarification of the topology of JEV NS2A in the ER membrane is necessary for understanding the role of pTMS7 of JEV NS2A.

The differences in in vitro growth kinetics between the GI strain Mie/41/2002 and the GV strain Muar, NS1-3^Muar^, or NS2A^Muar^ were more notable in mouse neuroblastoma cells than in Vero cells. These results imply that JEV NS2A is involved in the tropism of JEV. The ability to bind to the ER membrane of the cells used in this study might differ between Mie/41/2002 and Muar or Muar NS2A-bearing recombinant JEV strains. Little is currently known regarding the cellular factors that interact with NS2A, and identification of the host-cell molecules that interact with flavivirus NS2A will facilitate elucidation of the role of NS2A in cell tropism. Several lines of evidence indicate that flavivirus NS2A plays a role in inhibiting host interferon (IFN) responses [32,33,34,35,36], and JEV NS2A also blocks the host antiviral response of IFN-induced double-stranded RNA-activated protein kinase during JEV infection [35], which suggests that the NS2A^166^ amino acid might affect the ability of JEV NS2A to interfere with the host immune response. However, Vero cells are IFN-deficient and Neuro-2a cells infected with JEV do not secrete IFNβ1 [46], indicating that type I IFN-dependent signaling is blocked in both Vero cells and Neuro-2a, and that signaling pathways downstream of type I IFN may not be associated with the difference between the Muar and Mie/41/2002 strains. Activating transcription factor 3 (ATF3), which is induced by the infection of JEV, inhibits cellular antiviral signaling in Neuro-2a cells [46], suggesting that the ATF3 induction level might differ between Muar- and Mie/41/2002-infected Neuro-2a cells. In contrast to the growth data obtained in Vero and Neuro-2a cells, our preliminary data indicated that NS1-3^Muar^ v1 grew faster than NS1-3^Muar^ p10 in human lung cancer A549 cells, which retain intact signaling pathways related to the innate immune response (unpublished data). Further analysis of several cell types will be necessary to clarify whether the growth properties of Muar are specific to mouse neuronal cells. In this study, we examined viral growth by measuring the amount of infectious virus secreted into the culture medium. Analyses of the viral RNA and infectious particles in the JEV-infected cells will also be required to understand the different effects of NS2A on the growth of Muar and Mie/41/2002.

In addition, although both the NS1-3 and NS4A-5 regions are related to the low growth capacity of Muar in vitro (Figure 2), we only focused on the former region in this study. Analysis of the NS4A-5 region is also needed for a comprehensive understanding the growth nature and tissue tropism of JEV.

## Figures and Tables

**Table 1:** Amino acid residues that differ among amino acid sequences of NS1-3^Muar^ strains.

		Amino Acid at Position		
Category or Strain	Clone No.	prM^7^	prM^60^	NS2A^166^
Pin-sized-plaque clones	1	Gln	Lys	Tyr
	2	Gln	Lys	Tyr
	4	Gln	Lys	Tyr
	17 *	Gln	Lys	Tyr
	18	Gln	Asn	His
Native-sized-plaque clones	6	Arg	Asn/Lys mix	Tyr
	7 *	Arg	Asn	Tyr
	9	Gln/Arg mix	Lys/Asn mix	Tyr
	12	Arg	Asn	Tyr
	22	Arg	Asn	Tyr
NS1-3^Muar^ v1 *	-	Gln	Asn	His
NS1-3^Muar^ p10 *	-	Gln	Lys	Tyr
Muar	-	Gln	Asn	His
Mie/41/2002	-	Gln	Asn	Tyr

* Complete nucleotide sequences of these clones/viruses were determined in this study.

## Supplementary Materials

Available online at https://www.mdpi.com/1999-4915/12/7/709/s1. Table S1: Primers used for the construction of the recombinant JEVs, Figure S1: Comparison of amino acid sequences of JEV prM. GI: genotype I; GIII: genotype III; GV: genotype V, Figure S2: Comparison of amino acid sequences of JEV NS2A, Figure S3: Plaque morphology of NS1-3^Muar^ viruses passaged in Neuro-2a cells.
