# Supplementary material for: Amino Acid at Position 166 of NS2A in Japanese Encephalitis Virus (JEV) Is Associated with In Vitro Growth Characteristics of JEV
Source: Viruses. 2020 Jun 30;12(7):709. doi: 10.3390/v12070709 (PMC7412020; doi:10.3390/v12070709)
Supplement: Supplementary file 1 [file viruses-12-00709-s001.zip › Figure S1_200601pdf.pdf]

Figure S1

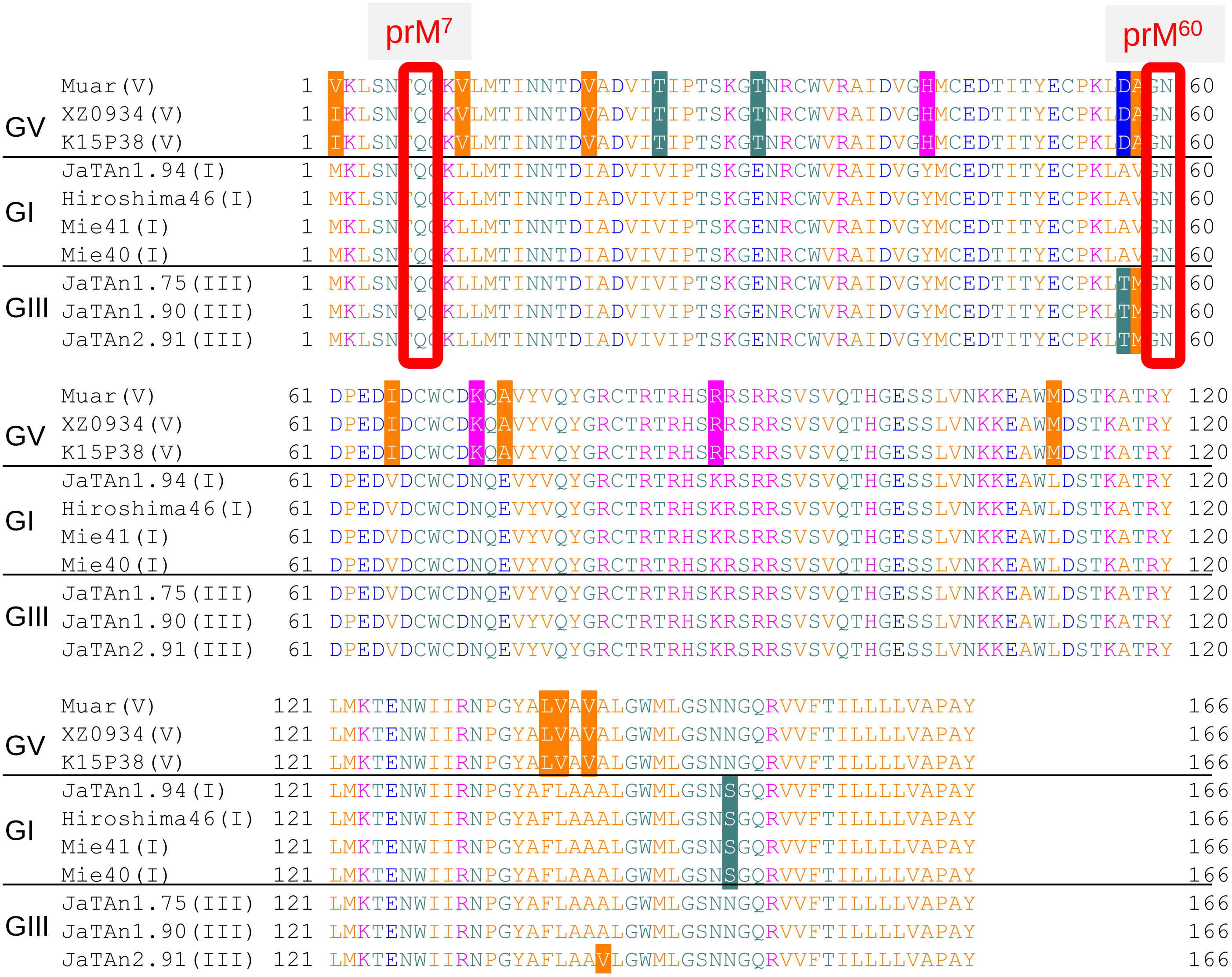

Figure S1. Comparison of amino acid sequences of JEV prM. GI: genotype I; GIII: genotype III; GV: genotype V.
