# Supplementary material for: Amino Acid at Position 166 of NS2A in Japanese Encephalitis Virus (JEV) Is Associated with In Vitro Growth Characteristics of JEV
Source: Viruses. 2020 Jun 30;12(7):709. doi: 10.3390/v12070709 (PMC7412020; doi:10.3390/v12070709)
Supplement: Supplementary file 1 [file viruses-12-00709-s001.zip › Figure S2_200622.pdf]

Figure S2

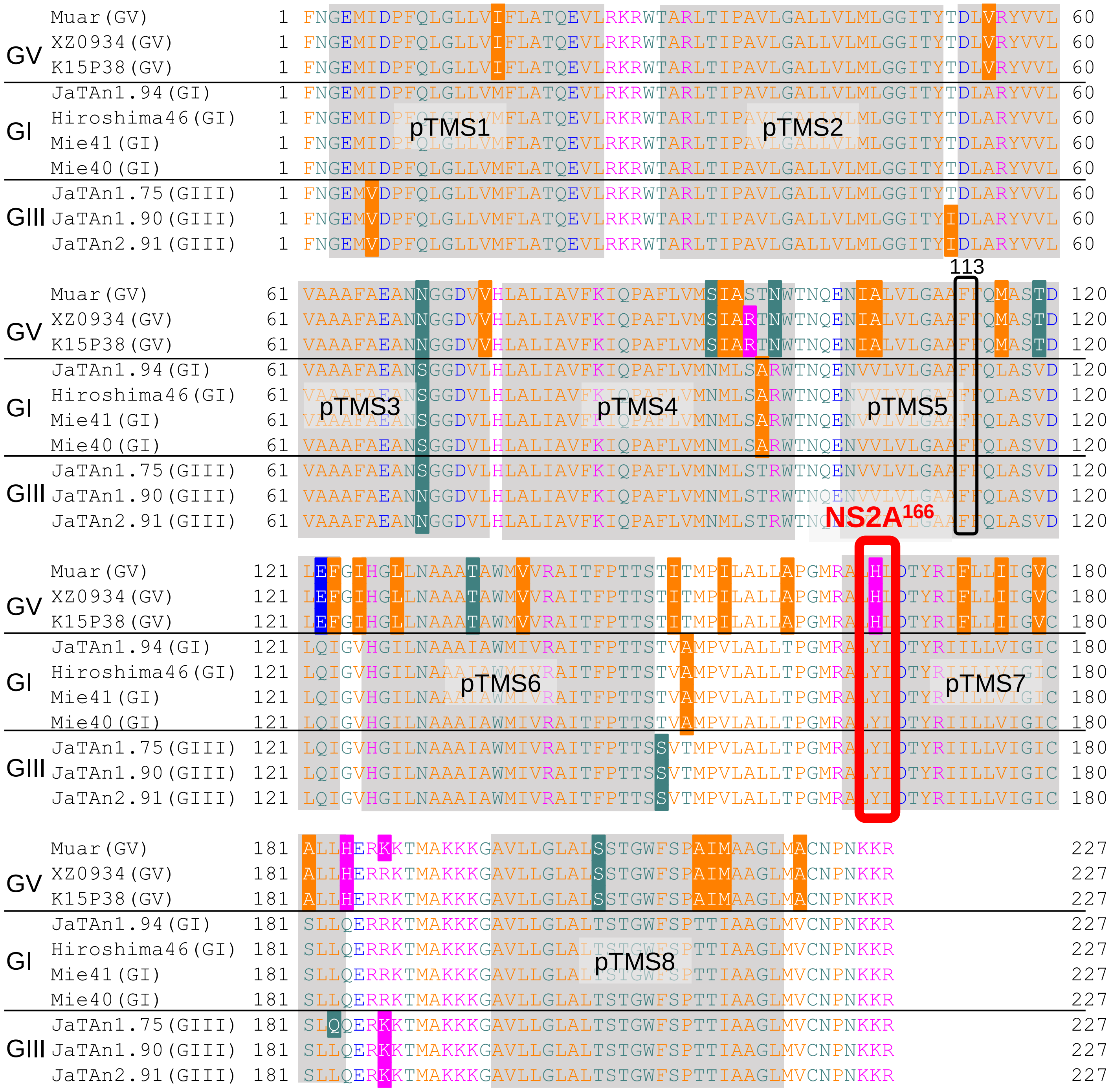

Figure S2. Comparison of amino acid sequences of JEV NS2A. Predicted transmembrane segments (pTMSs), indicated by light gray areas, were determined using SOSUI analysis (http://harrier.nagahama-i-bio.ac.jp/sosui). GI: genotype I; GIII: genotype III; GV: genotype V.
