# Supplementary material for: Amino Acid at Position 166 of NS2A in Japanese Encephalitis Virus (JEV) Is Associated with In Vitro Growth Characteristics of JEV
Source: Viruses. 2020 Jun 30;12(7):709. doi: 10.3390/v12070709 (PMC7412020; doi:10.3390/v12070709)
Supplement: Supplementary file 1 [file viruses-12-00709-s001.zip › Figure S3_200601pdf.pdf]

Figure S3

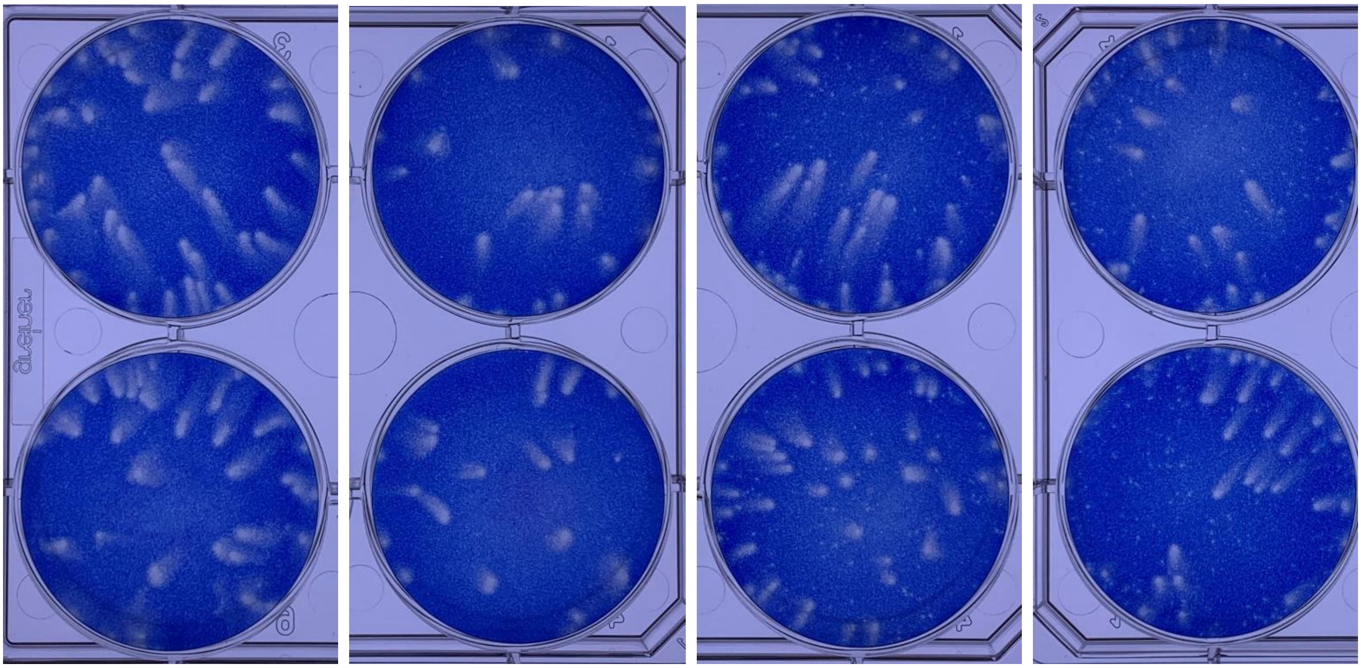

p3 p5 p7 p9

Figure S3. Plaque morphology of NS1-3^{Muar} viruses passaged in Neuro-2a cells; number of times viruses were passaged: 3 (p3), 5 (p5), 7 (p7), and 9 (p9).
