# Supplementary material for: Amino Acid at Position 166 of NS2A in Japanese Encephalitis Virus (JEV) Is Associated with In Vitro Growth Characteristics of JEV
Source: Viruses. 2020 Jun 30;12(7):709. doi: 10.3390/v12070709 (PMC7412020; doi:10.3390/v12070709)
Supplement: Supplementary file 1 [file viruses-12-00709-s001.zip › Table S1_200601pdf.pdf]

Table S1. Primers used for the construction of the recombinant mutant JEVs

Name	Sequence (5' - 3')	Recombinant viruses
JEV.NI-T7	GATCGCGGCCGCTAATACGACTCACTATAGAGAAG	5NCME^{Muar}
JE(GV)937Mlu.r	ACCACGCATTGGCCGTTGTTGCTGCCA	5NCME^{Muar}
J(GIGV)2398(InF)f:	AACGCACGAGACCGGTCGATCGCACTGGCTTTCTT	NS1-3^{Muar}
J(GIGV)6507(InF)r:	CTCAGGCATGCGGCCAAGCACCTCTAGAAAGCT	NS1-3^{Muar}
J(GIGV)6482(InF)f:	TAGAGGTGCTCGGCCGCATGCCGGAACATTTCATGG	NS4A-5^{Muar}
J(GIGV)9171(InF)r:	ATTTAGAAATCCTAATGCCTCAAATTCCAGGTAGCG	NS4A-5^{Muar}
J(GIGV)9136(InF)f:	GAGTTCGAAGCCTTAGGGTTCTTGAACGAGGACCAT	NS5-3N^{Muar}
J(GIGV)10965(InF)r:	ACTCTAGAGGATCCATGCATAGATCCTGTGTTCTTCCCCAC	NS5-3N^{Muar}
MuM41_2A.f	TCACAAGTCAATGCATTCAACGGCGAAATGATTGAC	NS1^{Muar}
M41Mu_NS1.r	CATTTCGCCGTTGAATGCATTGACTTGTGACTTCAC	NS1^{Muar}
MuM41_NS1.r	CATCTCCCCATTGAAAGCGTCAACCTGTGATCTAAC	NS2A^{Muar}
M41Mu_2A.f	TCACAGGTTGACGCTTTCAATGGGGAGATGATTGAT	NS2A^{Muar}
M41Mu_2A.r	GGTGGCTGGCCACCCTCTTTTCTTGTTTGGGTTGCA	NS2A^{Muar}
MuM41.2B.f	CCAAACAAGAAAAGAGGGTGGCCAGCCACCGAGTTC	NS2A^{Muar}
M41Mu_2B.f	CCAAACAAGAAGAGAGGATGGCCAGCGACAGAATTC	NS2B-3^{Muar}
MuM41_2A.r	TGTCGCTGGCCATCCTCTCTTCTTGTTTGGGTTGCA	NS2B-3^{Muar}
JEV(M41)4024f_4030C	GCCTTGCATCTGGACACTTAC	NS2A^{Y166H}
JEV(M41)4038r_4030G	GTCCAGATGCAAGGCCCTCAT	NS2A^{Y166H}
JEV(Mu)4023f_4029T	GCTCTTTATCTCGACACCTAC	NS2A$^{Muar-H166Y}$
JEV(Mu)4037r_4029T	GTCGAGATAAAGAGCTCTCAT	NS2A$^{Muar-H166Y}$
